# IL-6 Signaling Link between Inflammatory Tumor Microenvironment and Prostatic Tumorigenesis

**DOI:** 10.1155/2022/5980387

**Published:** 2022-04-12

**Authors:** Cosmin-Victor Ene, Ilinca Nicolae, Bogdan Geavlete, Petrisor Geavlete, Corina Daniela Ene

**Affiliations:** ^1^“Carol Davila” University of Medicine and Pharmacy, Romania; ^2^“St. John” Clinical Hospital of Emergency, Romania; ^3^“Victor Babes” Clinical Hospital of Infectious and Tropical Diseases, Romania; ^4^“Carol Davila” Clinical Hospital of Nephrology, Romania

## Abstract

Benign prostatic hyperplasia and prostate cancer are tumoral pathologies characterized by the overexpression of inflammatory processes. The exploration of tumor microenvironment and understanding the sequential events that take place in the stromal area of the prostate could help for an early management of these pathologies. This way, it is feasible the hypothesis that normalizing the stromal environment would help to suppress or even to reverse tumor fenotype. A number of immunological and genetic factors, endocrine dysfunctions, metabolic disorders, infectious foci, nutritional deficiencies, and chemical irritants could be involved in prostate tumor development by maintaining inflammation, affecting local microcirculation, and promoting oxidative stress. Inflammatory processes activate hyperproliferative programs that ensure fibromuscular growth of the prostate and a number of extracellular changes. Acute and chronic inflammations cause accumulation of immunocompetent cells in affected prostate tissue (T cells, macrophages, mastocytes, dendritic cells, neutrophils, eosinophils, monocytes). Prostate epithelial and stromal cells, peri-prostatic fat cells, prostatic microvascular endothelial cells, and inflammatory cells produce cytokines, generating a local inflammatory environment. Interleukin-6 (IL-6) proved to be involved in the prostate tumor pathogenesis. IL-6 ability to induce pro- and anti-inflammatory responses by three mechanisms of signal transduction (classical signaling, transsignaling, cluster signaling), to interact with a diversity of target cells, to induce endocrine effects in an autocrine/paracrine manner, and the identification of an IL-6 endogenous antagonist that blocks the transmission of IL-6 mediated intracellular signals could justify current theories on the protective effects of this cytokine or by alleviating inflammatory reactions or by exacerbating tissue damage. This analysis presents recent data about the role of the inflammatory process as a determining factor in the development of benign and malign prostate tumors. The presented findings could bring improvements in the field of physiopathology, diagnosis, and treatment in patients with prostate tumors. Modulation of the expression and activity of interleukin-6 could be a mean of preventing or improving these pathologies.

## 1. Prostatic Microenvironment

The tumor microenvironment forms a biological barrier around the tumor, which protects the tumor from the action of the host's defense mechanisms or the action of antitumor drugs. The prostate tumor microenvironment should be considered as a dynamic structure, consisting of extracellular matrix (three-dimensional network of collagen, fibronectin, sialoproteins, laminin, osteocalcins, cadherins, osteonectins, and vitronectins), constituent cells (endothelial cells, fibroblasts, myofibroblasts, and immune and mesenchymal cells), and soluble factors (cytokines, chemokines, growth factors, metalloproteinases) [1, 2]. A characteristic of the prostate tumor microenvironment is that it contains the same elements as the normal microenvironment, but with specific properties that promote inflammation (benign enlargement, hyperplasia, prostatic intraepithelial neoplasia) [3, 4].

## 2. Chronic Inflammation and Prostatic Tumorigenesis

Inflammation is considered the key in BPH development in latest clinical and experimental studies. BPH is a chronic, slow-progressing disease present in most men over 50 years, characterized by growth of prostate epithelial and stromal cells, which can cause urinary tract blockage and reduced urinary flow intensity. The urinary function impairment predisposes men with benign prostate hyperplasia to an increased risk of urinary tract infection and acute urine retention [5]. Meanwhile, the prostate cancer is less symptomatic because of its peripheral development, having a 97% of cases of adenocarcinoma and 3% of other tumor types. However, the inflammation plays a key role in the evolution of the disease, being correlated with the tumor aggressivity [6].

**Figure 1:**
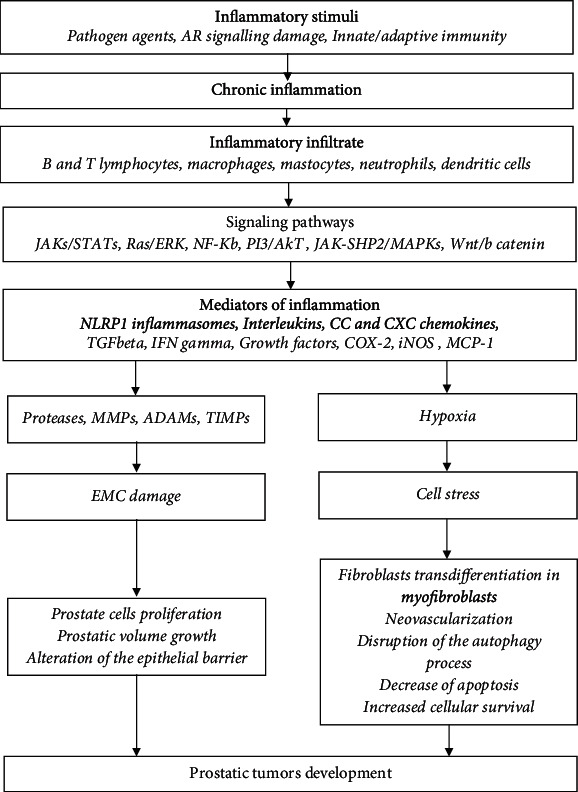
The inflammation role in prostate tumor development and evolution. AR-, JAK-, STAT-, ERK-, NF-*κ*B-, PI3-, Akt, SHP, MAPK, NLRP, CC, CXC, TGF, IFN, COX, iNOS, MCP, MMP, ADAM, TIMP, EMC.

Although prostatic tumor pathogenesis is not fully known, most recent studies suggest that a better understanding of the factors that contribute to the inflammatory response regulation will provide more information to the pathogenesis of prostatic tumors thus, contributing to the development of new therapeutic strategies. Inflammatory processes activate hyperproliferative programs that ensure the creation of a suitable microenvironment for the initiation and progression of tumors. There are a lot of speculations regarding the signaling of inflammation and the activation of specific and nonspecific immune defence mechanisms in the prostate tissue (Figure 1).

Infectious agents, urinary reflux, nutritional deficiencies, metabolic syndrome, ageing process, alkaloids in tobacco, cigarette smoke, and activation and assembly of inflammasomes were considered triggers for alteration of prostatic immune system via different molecular pathways, involving the development of inflammatory infiltrates [5–13]. Researching the relationship between smoking and sex steroid hormone in prostatic pathology showed impaired metabolism of sex steroid hormones and high levels of inflammation in prostatic tissue [13].

In the inflamed prostatic tissue, B and T activated lymphocytes, macrophages, mastocytes, neutrophils, dendritic cells, and monocytes were detected. Those activated cells produce cytokines (IL-1 alpha, IL-1 beta, IL-6, IL-8, IL-18), chemokines, growth factors, IFN gamma, TGF beta, and MCP-1 which provide fibromuscular growth of the prostate via STAT-1/NF-*κ*B signaling [5–8, 10, 11, 13–16]. Prolonged persistence of cytokines in the affected prostate tissue determines chronic inflammation, mediated by JAKs/STATs, MAPKs, and PI3/Akt [5, 17, 18]. Inflammatory factors induce the expression of COX-2 and iNOS that leading to growing the prostate cell proliferation rate [5, 15, 16, 19]. Local hypoxia could play a role as an inflammation mediator, developing neovascularization and differentiation from fibroblasts to myofibroblasts and some extracellular changes [19–21]. In affected prostate tissue, the activity of some proteolytic enzymes is overregulated, while some active substances are released in the intercellular spaces [22]. In this process, free radicals of oxygen and nitrogen are produced in excess and maintain chronic inflammation and induce oxidative degradation of biomolecules, disorganization of the extracellular matrix (EMC), and degradation of cellular architecture [1, 2, 12, 17–19]. All these phenomena lead to the proliferation of prostate cells, increased prostate volume, alteration of the epithelial barrier, urinary obstruction, vascularization disorder, apoptosis, cellular survival, and autophagy process (Figure 1).

## 3. Interleukin-6 Biologic Activities

A number of recent findings support the central role of IL-6 in prostate tumor pathology. The functional characteristics of IL-6 and its specific receptor (IL-6R) determine various biological responses in prostate tumors, probably explained by IL-6's ability to promote pro- and anti-inflammatory responses [7, 8, 23, 24], to interact with a diversity of target cells [10, 25–27], to induce autocrine and paracrine effects in prostate tissue [12, 24, 28], and to activate intracellular signaling pathways (Jak/S) [10];

IL-6 promotes three mechanisms for transmitting intracellular signals [7, 10, 25–27], being known an IL-6-induced signal buffer system [7, 8, 25, 26]; all the reactions modulated by IL-6 being produced in waterfall [7, 29].

IL-6 is a pleiotropic mediator, with multiple effects in the host's immune including inflammatory responses to different stimuli [7, 8, 10]; it plays the role of an endogenous pyrogen factor, triggering the fever process [7]. IL-6 is active in very low concentrations, participating in synergistic and antagonist processes, in the elaboration of the body's response to stress caused by inflammation [7, 8]. IL-6 secretion is fast and short-lived in an inflammatory, autoimmune, or oncogenetic process.

### 3.1. IL-6 and Its Receptors

IL-6 cytokine is synthesized as a precursor of 212 amino acids, with a molecular weight of 21.5-28.0 kDa depending on O- and N-glycosylation grade. IL-6 is mostly secreted by macrophages and also by T and B lymphocytes, monocytes, fibroblasts, endothelial cells, keratinocytes, synoviocytes, condrocytes, epithelial cells, mesangial cells, podocytes, astrocytes, stromal cells, adipocytes, and malignant cells. Thus, IL-6 is produced in response to infections, inflammation, trauma, autoimmune processes, and tumorigenesis [25, 30, 31]. IL-6 secretion is stimulated by endotoxins, cytokines, and microorganisms. Some proteins and microRNA control IL-6 synthesis at transcriptional and posttranscriptional levels. IL-6 mRNA formation is stimulated by NF-IL6 (nuclear factor of IL-60), Tax (transactivator protein), TAT (transactivator of the transcription), HBVX (hepatitis B virus X protein), Ahr (aryl hydrocarbon receptor), GR (glucocorticoid receptor), ER (estrogen receptor), Rb (retinoblastoma), and PPAR*α* (peroxisome proliferator–activated receptor *α*) and inhibited by microRNA (miR-155, miR-146a/b, miR-223). A group of factors provide IL-6 mRNA stability: ORF (open reading frame), p38, and arid5a; IL-6 mRNA complex disintegration: TTP (tristetraprolin), BRF (butyrate response factor), miR-365, and miR-608 [30, 32].

The specific receptor for IL-6 (IL-6R) is a membrane protein complex composed of two structural and functional subunits: a specific ligand subunit (IL-6R*α*) and a signaling subunit (gp130). The IL-6R*α* subunit is 80 kDa *α* chain, noted mMIL-6R or CD126. The gp130 signal subunit is a 130kYes b chain, noted CD130, the common component of several receptors, such as those linked to IL-11, IL-21, and IL-31. The active IL6-R receptor is on the surface of a limited number of cells (some lymphocytes, hepatocytes, neutrophils, monocytes/macrophages, and podocytes), while gp130 is ubiquitous on the surface of cell membranes. Both IL-6R*α* and gp130 can be cleaved, with the rapid appearance in circulation of soluble protein components, noted sIL-6R and sgp130 [25, 30, 31].

IL-6R soluble receptors are considered regulators of cytokine signaling and inflammatory events. Soluble IL-6 receptors (sIL-6R) have been identified in blood and urine. In humans, two distinct mechanisms induce sIL-6R secretion: the mIL-6R proteolytic splitting and mRNA IL-6R alternative splicing. The proteolytic cleavage of sIL-6R is activated of metalloproteinases (ADAM-10, ADAM-17) and serine proteases derived from neutrophils (cathepsin G, proteinase 3, neutrophilel elastase, neutrophil serine protease 4) [25, 29, 31]. Soluble glycoprotein sgp130 has been described as a specific inhibitor of IL-6-mediated signaling. It is found naturally in plasma. In humans, sgp130 is generated mainly by alternative splicing and not by proteolytic splitting [25, 26].

### 3.2. IL-6 Signaling Pathways

#### 3.2.1. IL-6 Classical Signaling

The classical signaling pathway (cis-signaling) mediated by IL-6 is functional in cells expressing CD126 and CD130. In cells that respond directly to IL-6 (macrophages, neutrophils, T helper cells, podocytes, hepatocytes), it binds to mIL-6R*α*. This complex interacts with the homodimer consisting of two subunits gp130 and initiates the transduction of the signal. Intracellular signaling is carried out by the Janus Kinase-Signal transducer and transcription activator (JAK-STAT), mitogen-activated kinase protein (MAPK), and phosphatidylinositol kinase 3/Akt kinase (PI3-AKT) [14, 27]. Though, IL-6 induces intracellular overregulation of cytokine signaling suppressor (SOCS-3) transcription genes by STAT3. This mechanism mediated by IL-6 is the classical signaling pathway.

The attachment of IL-6 to mIL-6R is critical for inducing the activation of transcription factors—nuclear factor-kappa B (NF-*κ*B), C/enhancer binding protein beta (C/EBP beta), CCAAT/enhancer binding protein delta (C/EBPdelta), cAMP responsive element binding protein 1 (CREB1), Jun D proto-oncogene (junD), v-Fos FBJ murine osteosarcoma viral oncogene homolog (c-Fos), and Jun oncogene (c-Jun)—depending on cell type and ligand specificity (Figure 1). Classical signaling induces in hepatocyte synthesis of acute phase proteins, proteins with anti-inflammatory and washable properties for immune defense. IL-6 classical signaling involved cell survival and proliferation regulation, epithelial cell regeneration, antiapoptotic signaling, and mitosis development [7, 10, 25, 27, 31, 33].

#### 3.2.2. IL-6 Transsignaling

The alternative signaling path (transsignaling) mediated by IL-6 requires the formation of a hexamer in which IL-6, sIL-6R, and gp130 interacts in a 2 : 2 : 2 [14] stochiometry. In the extracellular environment, sIL-6R binds to IL-6. This activated IL-6/sIL-6R complex interacts whether with the ubiquitous gp130 on the cell membrane or with circulant sgp130. IL-6/sIL-6R complex membrane-gp130 link is the alternative signaling pathway that allows IL-6 to modulate a wide spectrum of target cells and is called transsignaling and also for inhibiting IL-6 activity. Transsignaling is regulated by proinflammatory cytokines (IL-1 beta, TNF alpha), bacterial toxins, cell cholesterol depletion, PKC agonists, protease inhibitor, and degradated nucleic acids.

Transsignaling was described in inflammatory and autoimmune processes, lately being studied in cancer. The alternative signaling path mediated by IL-6 results in the regulation of neutrophil-monocyte transition, blocking the apoptosis of T cells and reducing the differentiation of Treg cells. In some situations, transsignaling produces protective effects by alleviating inflammatory reactions, and in other conditions, it exacerbates tissue destruction, by blocking inflammatory reactions [8, 10, 14, 31]. IL-6 transpresentation (cluster signaling) involves firstly IL-6 and IL-6R interaction, a complex located on cell membrane and secondly the link with sgp130 subunit, expressed on others cell membrane [7, 34]. The IL-6 signaling modulation was well documented in the latest studies [7].

The role of endogenous inhibitors in IL-6 signaling has been investigated in various biological systems. Endogenous negative feedback adjusters of the IL-6 signal can be grouped into as follows:

- *STAT Protein Inhibitors*. Prevent STAT-DNA interaction by blocking STAT dimerization
- *SH2-Protein Tyrosine Phosphatases*. Modulate tyrosine residue dephosphorylation, critical elements for kinases activation
- *Intracellular Suppressors of IL-6 Signaling (SOCS-1, SOCS-3)*. Inhibit JAK activation and disrupt cell cycle and apoptosis [35]

**Figure 2:**
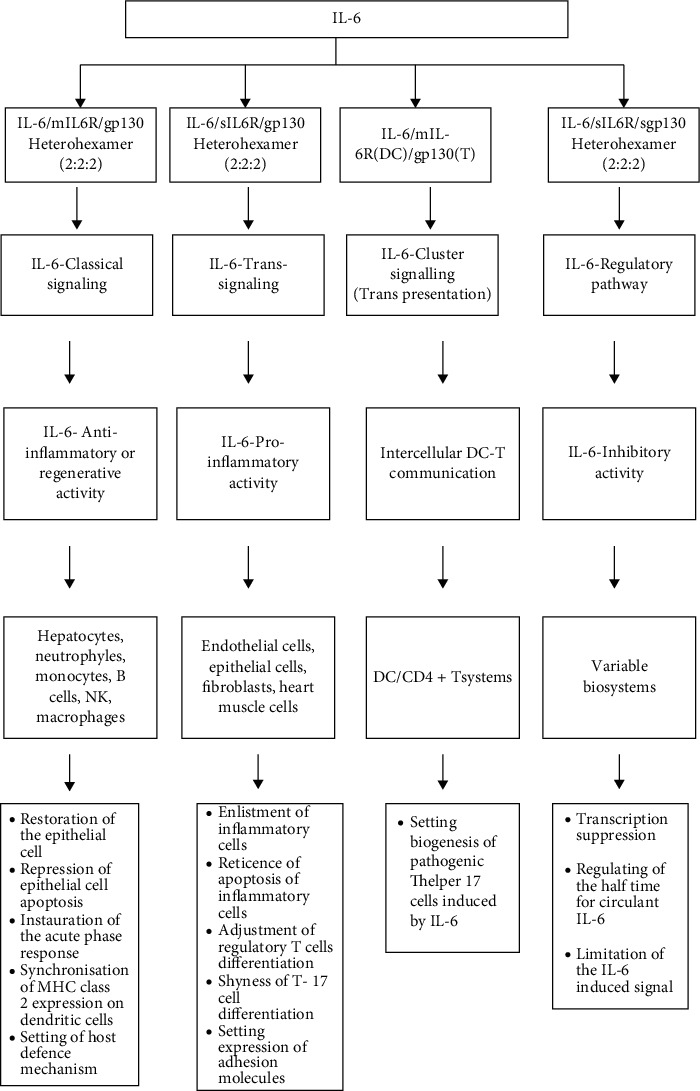
Schematic illustration of IL-6 signaling. IL-interleukin; IL-6R-IL6 receptor; mIL-6R-membranar-IL-6R; gp130-glycoprotein; sIL-6R-soluble IL-6R; sgp130-soluble gp130; mDC-dendritic cell membrane (transmitting cells); T-T cells (received cells).

Special attention was paid to the sgp130 subunit, extracellular suppressor of IL-6-induced signal. The circulating protein sgp130 is in competition with membrane gp130 for linking with IL-6/sIL-6R complex. IL-6 classical-mediated signaling via membrane IL-6R receptors is not affected by circulating levels of sgp130. An excess of sgp130 leads to competitive in vivo inhibition of IL-6/sIL-6R complex. Thus, sgp130 is considered a natural antagonist of IL-6/sIL-6R complex that can prevent systemic transsignaling and cluster signaling within inflammatory diseases [7, 8, 14, 25–27] (Figure 2).

Recently, it has been reported that sIL-6R to sgp130 serum ratio could be associated with overexpression of a particular IL-6 signaling pathway [8]. When the amount of sgp130 is greater than sIL-6R, the simultaneous activation of classical signaling and IL-6-mediated transsignaling takes place. When the amount of sIL6R is greater, IL-6-mediated transsignal is overexpressed. Consequently, pharmacological modulation of the sIL-6R/sgp130 ratio could prevent excessive organ damage during active inflammatory events [8].

### 3.3. IL-6 and IL-6R in the Prostate

#### 3.3.1. IL-6 and IL-6R Expression in Prostate Tumours

IL-6 is secreted by prostate cells, periprostatic fat cells, prostate microvascular endothelial cells, and inflammatory and immune cells recruited into the assaulted tissue microenvironment [26, 27, 31]. IL-6 and its receptor expression was investigated in normal prostate tissue, benign and malignant prostate lesions, slow-growing androgen-sensitive prostate cell lines (LNCaP), and rapid-growth androgen-insensitive prostate cell lines (DU145, PC3) [28, 35, 36].

In normal prostate tissue, IL-6 was immunolocalized mostly in basal epithelial cells and poorly expressed in stromal cells, while gp130 was detected only in stromal cells [27]. IL-6R has been identified in epithelial and stromal cells [35]. The most important source of IL-6 in prostate tissue is macrophages/monocytes, being involved in the inflammatory response, though, secreting cytokines and MMPs [14, 26]. Monocytes, macrophages, and neutrophils express IL-6R, which ensures the functioning of the classical IL-6-mediated signaling pathway. In benign prostate hypertrophy, IL-6 was preferentially immunolocated in basal epithelial cells, and gp130 was limited to epithelium and stroma [27].

In malignant prostate tissue, IL-6 is intensely expressed in adenocarcinoma, being secreted preferentially by glandular cells, and its level is elevated in patients with a low prognosis. IL-6 was detected in all cell types, and its immunostability increased with the degree of Gleason. The gp130 subunit was detected in stroma and epithelium, and its expression increased with Gleason grade [27]. IL-6R receptors were intensely expressed in malignant prostate cells [27]. The IL-6/IL-6R signaling pathway could be considered a molecular marker associated with the progression of prostate cancer. Serum concentrations of IL-6 and sIL-6R were correlated with advanced stages of the disease and a poor prognosis in prostate cancer patients [27].

#### 3.3.2. IL-6 and IL-6R Synthesis Regulation in Prostate Cancer

Regulation of IL-6 synthesis and secretion in prostate cancer is the result of several cellular processes, some of which being interconnected. One interesting connection was TGF beta—IL-6. Serum TGF beta was overexpressed in prostate cancer patients, and it increased IL-6 expression. TGF beta acted as an inhibitor of prostate cancer growth in vitro. In vivo, TGF beta activated the angiogenic cascade, suppressed the immune responses, and induced the expression of matrix metalloproteinases, though causing tumor growth. Though, IL-6 production might stimulate angiogenesis [27].

IL-6 study in LNCaP-IL-6+ cells culture showed that IL-6 production might be a consequence of reduced retinoblastoma protein expression. It is also important to note that the presence of andrographolide inhibited IL-6 expression. These findings are consistent with the inhibition of NF-*κ*B activity by dihydrotestosterone. PC-3 and DU-145 AR-negative cells secreted high levels of IL-6. IL-6 production was stimulated by JunD and protein kinases [35]. In LNCaP, DU145, and PC3 cell lines, this cytokine and its specific receptors were widely expressed, but they were absent in normal prostate epithelial PZ-HPV-7 culture cells [27]. DU145 and PC3 cell lines proliferated in response to IL-6 stimulation. As a result, IL-6 acted as an autocrine and/or paracrine proliferative factor in prostate cell lines [28].

There is evidence that IL-6 acts as a paracrine growth inhibitor under certain conditions and as an autocrine growth stimulant in other situations. IL-6 might have different functions in the proliferation of prostate cell lines according to the phenotypic characteristics of the cells and the microenvironment of the culture system [27, 28]. Prostate microvascular endothelial cells synthesize and secrete IL-6 in a paracrine manner, under the induction of IL-1, LPS, TNF alpha, and IL-4. IL-6 modulates processes such as andrographolide subregulation, leukocyte recruitment at the site of inflammation, chemokine secretion, expression of endothelial surface adhesion molecules, neovascularization and angiogenesis, and metastasis. Endothelial cells do not express IL-6R, so IL-6 regulates endothelial function only through transsignaling, being involved in multiple biologic processes in prostate tumors [24, 31].

### 3.4. Biologic Processes Mediated by IL-6 in Prostate Tumors

The inflammatory response in prostate is ensured by the active complex IL-6/IL-6R. The involvement of IL-6 in benign prostatic hyperplasia is complex. IL-6 has multiple, distinct, or even contradictory pathophysiological effects in benign prostatic hyperplasia. At this level, IL-6 is secreted by prostate cells, periprostatic fat cells, prostate microvascular endothelial cells, and inflammatory and immune cells recruited into the aggressed tissue microenvironment [26, 27, 31].

In chronic inflammatory diseases, IL-6 appears to be a reliable indicator of future inflammatory reactions [14, 37, 38]. IL-6 mainly modulates the body's immune and inflammatory responses. IL-6 exerts proinflammatory effects, being synthesized in response to infections, inflammation, or trauma. Cytokine IL-6 is considered the most important inducer of hepatic production of acute phase reactants: fibrinogen, serum amyloid A, haptoglobin, C-reactive protein, complement, and hepcidin [32].

IL-6/IL-6R modulated innate and adaptive immunity in the prostate. IL-6/ILs-6R was involved in regulation of acute inflammatory responses (chemokine production, T-helper 2 cell cytokine production, leucocyte chemotaxis) and immune processes (neutrophil-mediated immunity, immunoglobulin secretion, hepatic immune response) in cell activation and proliferation (B cell activation, T-cell proliferation and differentiation, platelet activation, osteoblast differentiation), in apoptotic processes, in DNA replication, in signaling pathways (ERK1 and ERK2 cascade, JAK/STAT cascade, MAPK cascade, cytokine and chemokine mediated signaling pathway, activation of NF-kappaB transcription factor, peptidyl-serine and peptidyl-tyrosine phosphorylation), in gene expression, and in angiogenesis (VEGF production) [5, 23, 25, 30]. The cytokine IL-6 stimulated humoral and cellular immune responses by acting on both B and T lymphocytes, promoting their growth and differentiation. IL-6 was involved in regulating the differentiation of CD4 + lymphocytes into regulatory (Treg) and helper17 (Th17) T cells. IL-6 and TGF beta triggered Th17 differentiation by increasing ROR expression and attenuating Treg generation by STAT3. Th17 cells secreted proinflammatory cytokines and initiated inflammatory responses. IL-6 modulated apoptosis by producing IL-2 and activating STAT3 and activated the generation of Th2 cytokines via the C/EBP transcription factor. IL-6 was involved in B cell-induced inflammation through follicular Th cells. As a result, IL-6 plays a key role in the prostate-mediated immune response at the prostate level [27, 31].

#### 3.4.1. Biologic Processes Mediated by IL-6 in Prostate Hypertrophy

An important role in BPH development and progression is played by inflammation. Histopathological examination of BPH showed infiltration of lymphocytes and macrophages around the glandular area, cells that secreted high levels of IL-6 and CXCL8, secondarily, with epithelial and stromal cell growth [5]. Thus, IL-6 was produced in the presence of systemic insults (bacterial and viral infections, inflammation, trauma, altered AR signaling, hypoxemia, toxins, oxidized lipids, advanced glycosylation end products), quickly reaching detectable serum levels. IL-6 regulated macrophage/monocyte/dendritic cell differentiation, by triggering and stimulating the expression of M-CSF receptors (on monocytes), complement receptors, Fc, and F4/80 receptors (on macrophages), via JAK/STAT signaling. Moreover, IL-6 stimulated the in vivo and in vitro expression of the genes Jun-C, Jun-B, Jun-D, Jak, Egr, lysozyme, ferritin (on macrophages), and MCP-1 (on monocytes) [26, 27, 31, 39].

Clinical and laboratory data showed a statistically significant positive correlation between IL-6 and prostate volume and a weak positive correlation with IPSS (International Prostatic Symptom Score). During treatment with dutasteride, IL-6 showed a progressive decrease in patients with BPH [37]. The positive, statistically significant, correlations between the acute phase reactants and clinical elements such as IPSS, PVR (postvoiding residue), and prostate volume sustain the role of inflammation in BPH development. The acute phase positive proteins (CRP, ferritin, and ceruloplasmin) were characterized by elevated serum values and a positive correlation with the IPSS score and prostate volume, while the negative acute phase proteins (transferrin and albumin) showed decreased levels in BPH patients. In patients with BPH, IL-6 regulated the status of iron and zinc. In condition of high inflammation and high levels of IL-6, the liver decreased the synthesis of albumin, fibronectin, ferroportin, ZIP14, and transferrin, in turn initiating liver regeneration processes [32].

IL-6/IL-6R regulated the cellular response to infectious agents (bacteria, fungi, protozoa, viruses) detected in BPH patients. IL-6 is involved in defense response to Gram negative and positive bacterium, to protozoa, and to virus. IL-6 also modulated the cellular response to antibiotics, glucocorticoids, insulin, estradiol stimulus, HGF, IL-1, TNF alpha, LPS, nutrient levels, and cytokines. Moreover, it has been suggested that chronic retention of T. vaginalis in the prostate is associated with BPH development [5, 33]. T. vaginalis-infected BPH-1 cells could induce inflammatory responses. In addition, T. vaginalis-stimulated BPH-1 cells produced proinflammatory cytokines and induce monocyte and mast cell migration. Though, increased IL-6 promoted the development of benign prostatic hyperplasia and prostate cancer [5, 11, 19, 33]. When BPH-1 cells and prostate epithelia were treated with IL-6, cell proliferation was increased. IL-6 activated intracellular signaling via JAK/STAT, Ras/ERK, or PI3K/Akt [32]. In T. vaginalis-infected BPH-1 cells, increased levels of IL-6 and high expression of JAK2 and phosphorus STAT3 were detected. Moreover, the treatment with a JAK2 inhibitor reduced IL-6 production. These results suggested that JAK2/STAT3 signaling was involved in the production of IL-6 [5, 33] in BPH patients. BPH-1 cells infected with T. vaginalis produced cytokines, such as CXCL8, CCL2, IL-1 *β*, and IL-6, through cellular signaling pathways involving ROS, MAPK, and NF-*κ*B. T. vaginalis infection in patients with BPH by the effects mentioned above could be responsible for the development of lower urinary tract symptoms [5, 33]. IL-6/IL-6R stimulated androgen hormone synthesis and AR expression. IL-6 functioned as a paracrine growth factor for LNCaP and as an autocrine growth factor for DU145 and PC3 but had no stimulatory effect on BPH-derived epithelial cells [28]. Some interesting studies sustained also IL-6 role in BPH development, by reducing dihydrotestosterone production after dutasteride treatment, increase of antioxidant capacity, reduction of prooxidant levels, and progressive reduction of serum IL-6 [37, 39].

#### 3.4.2. Biologic Processes Mediated by IL-6 in Prostate Cancer

The expression of IL-6 and IL-6R in prostate cancer, as well as the role of IL-6 as a growth factor in prostate cancer, was well documented. IL-6 protein levels were correlated with disease stages and high levels of IL-6 with poor prognosis. Some studies analyzed the autocrine and paracrine effects of IL-6 in IL-6 positive LNCaP cells. IL-6 induced the growth of neuroendocrine cells, overexpressed in castration-resistant prostate cancer (CRPC). IL-6 stimulated androgen and androgen receptor synthesis in prostate cancer cells. IL-6 was a potent inducer of the protein encoded by the S100P gene (S100P) that was upregulated in CRPC and metastatic prostate cancer and therefore was involved in regulating androgen sensitivity. Constitutive expression of IL-6 in the prostate, similar to chronic inflammation, activated STAT3, reprogrammed the transcription of a set of genes, activated IGF, and amplified inflammation in the prostate and periprostatic adipose tissue [26, 40–43].

Because IL-6 is a cytokine produced by many cell types in inflamed prostate tissue, numerous studies suggest a direct link between chronic inflammation and prostatic tumor development. In vivo and in vitro studies sustain the above findings by the following results results: activation of STAT3 in prostate stromal cells, infiltration of many types of inflammatory cells in the prostate, and the presence of inflammatory cells in peri-prostate adipose tissue. IL-6 is considered the best-known activator of STAT3. In normal prostate cells, STAT3 was inactive, and SOCS3 was overexpressed; SOCS3 interacted with gp130 and prevented IL-6 signaling and STAT3 activation. In chronic inflammation, STAT3 was activated by blocking the SOCS3 gene via hypermethylationand IL-6-mediated signaling was constitutively expressed [7, 10, 26, 40–43].

IL-6 also mediated a series of anti-inflammatory effects, like completing the inflammatory cascade by suppressing the IL-1 and TNF synthesis, simultaneously with the stimulation of IL-1R alpha synthesis in different types of cancer. The anti-inflammatory activity of IL-6 included the activation of STAT3, which involved the regeneration of epithelial cells and the induction of the acute phase hepatic response [7, 10, 25]. IL-6 had an anti-inflammatory role in myeloid cells. In these cell cultures, the exposure to IL-6 before stimulation with microbial products (e.g., lipopolysaccharide (LPS)) inhibited class II major histocompatibility complex (MHC) expression and proinflammatory mediators in bone marrow-derived dendritic cells. In fact, IL-6-deficient mice showed a higher-class II MHC expression on dendritic cells than that in wild-type mice, whereas mice with enhanced IL-6 signaling caused by the loss of the SOCS3-binding site in gp130 (F759 mice) showed a lower expression. IL-6 reduced the level of cystatin C, an endogenous inhibitor of cathepsins, thereby increasing cathepsin S activity and subsequent degradation of MHC class II components in IL-6-treated dendritic cells. Similarly, prolonged action of IL-6 had been shown to mimic the anti-inflammatory effects of IL-10, which also activated STAT3, in macrophages. Moreover, the anti-inflammatory effects of IL-6 were manifested in a murine model of allergic asthma. In this model, IL-6-deficient mice showed exaggerated lung inflammation whereas lung-specific overexpression of IL-6 reduced the disease symptoms. Importantly, IL-6 stimulation was also known to suppress T-cell-receptor-mediated signaling via SOCS3. Thus, direct IL-6 stimulation in certain immune cell populations could induce an anti-inflammatory signal in prostate cancer and hypertrophy [44–46].

Because IL-6 is a cytokine produced by many cell types in the inflamed hyperpasic prostatic tissue, a lot of studies suggested a direct link between inflammation and prostatic tumors. The regulation of the inflammasome activity at posttranslational levels (phosphorylation, ubiquitination, de-ubiquitination, proteolytic processing, *S*-nitrosylation, and ADP-ribosylation) [45]; regulation of NLRP1, caspase-1, and cytokines (IL-18 and IL-1*β*) [46]; and modulation of anti- and proinflammatory activities of IL-6 [5, 7, 14, 46] might offer innovative therapeutic targets for prostatic tumor-related inflammation.

## 4. Conclusions and Future Directions

The study of inflammation in prostate tumors is very actual and a continuously research area. Blocking inflammation could allow the identification of control factors that react quickly, and though, inflammation takes place within certain limits, allowing the body to regenerate its affected tissues. The ability of interleukin-6 to induce pro- and anti-inflammatory activities by using three mechanisms of information transduction (classical signaling, transsignaling, cluster signaling), the ability of interleukin-6 to interact with a variety of target cells to induce endocrine effects in autocrine/paracrine manner, and the identification of an endogenous antagonist that blocks the transmission of interleukin-6-mediated intracellular signals could justify current theories on the protective effects of IL-6 cytokine in prostate tumors, by attenuating inflammatory reactions in certain conditions or exacerbating tissue damage in other conditions. Antitumor therapeutic methods targeting the tumor microenvironment tend the inversion of the tumor microenvironment into normal microenvironment. Immunotherapy focused on IL-6 involvement in prostate tumor could help the urologists and oncologists for a better management of the patients with prostatic tumors.

## Data Availability

The studies used to support the findings of this study are available from the corresponding author upon request.

## Abbreviations

BPH: Benign prostate hyperplasia
IL-6: Interleukin-6
IFN gamma: Interferon gamma
TGF beta: Transforming growth factor beta
MCP-1: Monocyte chemoattractant protein-1
STAT-1: Signal transducer and activator of transcription 1
NF-*κ*B: Nuclear factor kappa B
JAKs: The Janus kinases
MAPK: Mitogen-activated protein kinase
PI3: Phosphoinositide 3-kinases
Akt: Protein kinase B
EMC: Extracellular matrix
AR: Androgen receptor
ERK: Extracellular signal-regulated kinases
NF-*κ*B: Nuclear factor kappa-light-chain-enhancer of activated B cells
SHP: Tyrosine phosphatase
NLRP: Nucleotide-binding oligomerization domain, leucine rich repeat and pyrin domain containing
CC: *β*-Chemokine
CXC: *α*-Chemokines
COX: Cyclooxygenase
iNOP: Inducible nitric oxide synthase
MMP: Matrix metalloproteinase
ADAM: A disintegrin and metalloproteinase
TIMP: Tissue inhibitors of metalloproteinase
NF-IL6: Nuclear factor of IL-60
Tax: Transactivator protein
TAT: Transactivator of the transcription
HBVX: Hepatitis B virus X protein
Ahr: Aryl hydrocarbon receptor
GR: Glucocorticoid receptor
ER: Estrogen receptor
Rb: Retinoblastoma
PPAR*α*: Peroxisome proliferator–activated receptor *α*
ORF: Open reading frame
p38: Protein kinase
arid5a: AT-rich interaction domain 5A
IL-6 mRNA: Complex disintegration
TTP: Tristetraprolin
BRF: Butyrate response factor
PI3-AKT: Phosphatidilinositol kinase 3/Akt kinase
SOCS-3: Cytokine signaling suppressors
C/EBP beta: C/enhancer binding protein beta
CCAAT: Enhancer-binding proteins
CREB1: cAMP responsive element binding protein 1
junD: Jun D proto-oncogene
c-Jun: Jun oncogene
mDC: Dendritic cell membrane (transmitting cells)
T-T cells: Received cells
LNCaP: Androgen-sensitive prostate cell lines
DU145, PC3: Rapid growth cell lines insensitive to androgen
PZ-HPV-7: Cellosaurus cell line
ZIP14: Zrt- and Irt-like–Zn transporter
CXCL8: C-X-C motif chemokine ligand 8
PVR: Postvoiding residue
EMP: Erythroblast macrophage protein
MHC: Major histocompatibility complex.
